# A Quantitative Approach to Unravel the Role of Host Genetics in IgG-FcγR Complex Formation After Vaccination

**DOI:** 10.3389/fimmu.2022.820148

**Published:** 2022-02-22

**Authors:** Melissa M. Lemke, Robert M. Theisen, Emily R. Bozich, Milla R. McLean, Christina Y. Lee, Ester Lopez, Supachai Rerks-Ngarm, Punnee Pitisuttithum, Sorachai Nitayaphan, Sven Kratochvil, Bruce D. Wines, P. Mark Hogarth, Stephen J. Kent, Amy W. Chung, Kelly B. Arnold

**Affiliations:** ^1^ Department of Biomedical Engineering, University of Michigan, Ann Arbor, MI, United States; ^2^ Department of Microbiology and Immunology, The University of Melbourne, at the Peter Doherty Institute for Infection and Immunity, Melbourne, VIC, Australia; ^3^ Department of Disease Control, Ministry of Public Health, Bangkok, Thailand; ^4^ Vaccine Trial Centre, Faculty of Tropical Medicine, Mahidol University, Bangkok, Thailand; ^5^ Armed Forces Research Institute of Medical Sciences, Bangkok, Thailand; ^6^ The Ragon Institute of Massachusetts General Hospital, Massachusetts Institute of Technology and Harvard University, Cambridge, MA, United States; ^7^ Immune Therapies Group, Burnet Institute, Melbourne, VIC, Australia; ^8^ Department of Immunology and Pathology, Monash University, Melbourne, VIC, Australia; ^9^ Department of Clinical Pathology, The University of Melbourne, Melbourne, VIC, Australia; ^10^ ARC Centre of Excellence in Convergent Bio-Nano Science and Technology, The University of Melbourne, Melbourne, VIC, Australia; ^11^ Melbourne Sexual Health Centre, Alfred Hospital, Monash University Central Clinical School, Melbourne, VIC, Australia

**Keywords:** systems serology, Fc receptor, IgG1 allotype, Fc receptor polymorphism, HIV, RV144, ADCC, vaccine boosting

## Abstract

Fc-mediated immune functions have been correlated with protection in the RV144 HIV vaccine trial and are important for immunity to a range of pathogens. IgG antibodies (Abs) that form complexes with Fc receptors (FcRs) on innate immune cells can activate Fc-mediated immune functions. Genetic variation in both IgGs and FcRs have the capacity to alter IgG-FcR complex formation *via* changes in binding affinity and concentration. A growing challenge lies in unraveling the importance of multiple variations, especially in the context of vaccine trials that are conducted in homogenous genetic populations. Here we use an ordinary differential equation model to quantitatively assess how IgG1 allotypes and FcγR polymorphisms influence IgG-FcγRIIIa complex formation in vaccine-relevant settings. Using data from the RV144 HIV vaccine trial, we map the landscape of IgG-FcγRIIIa complex formation predicted post-vaccination for three different IgG1 allotypes and two different FcγRIIIa polymorphisms. Overall, the model illustrates how specific vaccine interventions could be applied to maximize IgG-FcγRIIIa complex formation in different genetic backgrounds. Individuals with the G1m1,17 and G1m1,3 allotypes were predicted to be more responsive to vaccine adjuvant strategies that increase antibody FcγRIIIa affinity (e.g. glycosylation modifications), compared to the G1m-1,3 allotype which was predicted to be more responsive to vaccine boosting regimens that increase IgG1 antibody titers (concentration). Finally, simulations in mixed-allotype populations suggest that the benefit of boosting IgG1 concentration versus IgG1 affinity may be dependent upon the presence of the G1m-1,3 allotype. Overall this work provides a quantitative tool for rationally improving Fc-mediated functions after vaccination that may be important for assessing vaccine trial results in the context of under-represented genetic populations.

## Introduction

Antibodies (Abs) are a vital component of the protective immune response elicited by vaccination. Immunoglobulin G (IgG) Abs that activate Fc effector functions are important for protection against a number of pathogens (1–5) and have been correlated with protection in HIV vaccine trials (6, 7). Antigen bound IgG immune complexes can trigger Fc effector functions by the crosslinking of IgG Fc portions with Fc receptors on the surface of innate immune cells. Fc functional capacity is directly correlated to the number of immune complexes formed that activate Fc receptors (8), which is regulated by numerous factors including IgG subclass concentrations, availability of FcRs and their respective binding properties (9). These properties vary in individuals and several studies have demonstrated that they are influenced by genetic factors including IgG1 allotypes and FcR polymorphisms (10–12).

Currently, four human IgG1allotypes (G1m1 [or G1m(a)], G1m2 [or G1m(x)], G1m3 [or G1m(f)], G1m17 [or G1m(z)]) have been identified (13). These allotypic determinants are inherited in a Mendelian pattern, i.e. sets of G1m haplotypes are inherited. G1m3 and G1m17 allotypes are mutually exclusive and refer to different amino acid changes at the same position (14). G1m17 allotypes are almost always linked with G1m1 (written together as G1m1,17 but hereafter referred to as G1m1 in this text), whereas G1m3 can exist with or without G1m1 (e.g. G1m1,3 or G1m-1,3 respectively). Interestingly, common allotypes are shared within ethnic or genetic populations. People with African ancestory have an enriched prevalence of G1m1 allotypes, those with a European ancestory have enriched G1m1 and G1m-1,3 allotypes while those with Asian ancestory have enriched G1m1 and G1m1,3 allotypes (15, 16). Recent research suggests that IgG1 allotypic variation is linked with all four IgG subclass concentrations, potentially due to allotype-linked variation in expression and degradation (12). Importantly these allotype-linked differences in IgG subclass concentrations are also observed in an antigen-specific manner upon vaccination. For example a recent phase I HIV vaccine trial (17) observed that G1m1 vaccinees (G1m1 & G1m1,3) reported to have higher HIV-specific IgG1:IgG2 ratios compared to the G1m-1,3 allotype, mainly driven by elevated HIV-specific IgG1 titers in G1m1 individuals (10).

In parallel, a range of FcγR polymorphisms have been identified in humans, some of which have greater Fc binding affinity and hence are associated with enhanced Fc functional capacity (11, 18–20). Individuals carrying the high affinity FcγRIIa H^131^ polymorphism, most commonly associated with enhanced ADCP, have positive outcomes in both cancer (21) and infectious diseases, including HIV (22, 23). The FcγRIIIa V^158^ polymorphism, with higher affinity than FcγRIIIa F^158^, has been associated with enhanced ADCC functionality and linked to better outcomes within the mAb cancer field (24, 25). Conversely, this same polymorphism has been associated with HIV disease progression (26) and the lack of protection in the HIV VAX004 vaccine trial (27). The distribution of these polymorphisms can also vary between different populations (28). Though FcR polymorphisms clearly dictate affinity for IgG subclasses, their overall role in FcγR activation is more ambiguous, especially in the context of variability in IgG subclass concentrations.

To date, few studies have explored the relative roles of IgG1 allotypes and FcR polymorphisms in FcR activation after vaccination, as their distributions are not measured in vaccine trials. In addition, it is difficult to unravel the parallel influences of both subclass concentrations and binding affinities that arise from differences in IgG1 allotype and FcγR polymorphism combinations. Recently, we computationally assessed the mechanistic underpinnings of IgG-FcγR complex formation after vaccination and demonstrated that synergistic relationships can occur between antibody parameters that regulate FcγR activation, that would not be apparent from studying each in isolation (21). Therefore, it is plausible that multiple immunogenetic changes may also have synergistic influences upon FcγR activation, which are greater than those that would be expected from simply summing changes evaluated in isolation. These are often too complex to be captured experimentally when parameters are examined individually.

Here we use data from the HIV RV144 vaccine trial and a mechanistic computational model to assess the relative roles of IgG1 allotypes and FcγR polymorphisms in IgG-FcγRIIIa immune complex formation after HIV vaccination. We demonstrate how genetic background may influence an individual’s Fc functional response upon vaccination and suggest specific interventions that would most effectively improve IgG-FcγRIIIa immune complex formation in each allotype/polymorphism combination.

## Materials and Methods

**Figure 1:**
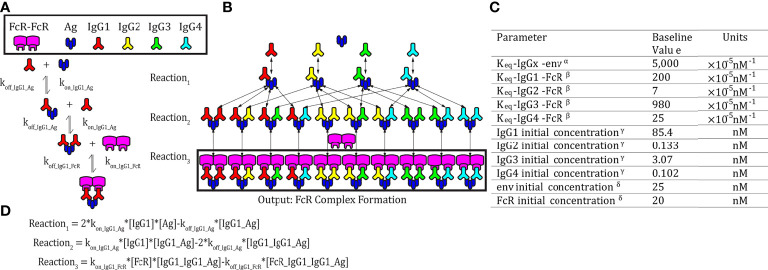
Model schematic. **(A)** An example set of reversible reactions describing the sequential binding of IgG1 to antigen (Ag) and dimeric FcγR with the respective forward (k_on_) and reverse (k_off_) reaction rates. **(B)** Ordinary differential equations were used to predict total HIV Ag-IgG-FcγR complexes formed as a function of concentration and binding affinity of Ag, IgG subclasses, and FcγR. The model assumes a single FcγR type. Reversible reactions are represented by double ended arrows. Model output was the sum of all dimeric FcγR complexes formed (boxed in black) at steady state. **(C)** The baseline parameters for FcγRIIIA-V^158^ complex formation with the following sources: ^α^SPR measurement from pooled purified IgG from HIV infected individuals binding to monomeric gp120. All IgG subtypes share one affinity value to the antigen of focus, gp120 env (unpublished data). ^β^K_eq_ measured in Bruhns et al. (11). ^γ^The average estimated IgG concentrations from individuals 1-30 in the RV144 data in this manuscript (see methods for notes on conversion from MFI to mM unit). ^δ^Concentrations used in multiplex experimental protocol. **(D)** Equations describing the example reactions in panel **(A)** Reactions follow mass action kinetics and consist of a forward reaction (on rate, k_on_, multiplied by the concentrations of substrates) and a reverse reaction (off rate, k_off_, multiplied by the concentration of the product of the forward reaction). Differential equations for change in each complex over time were generated for each complex.

We applied an ordinary differential equation (ODE) model as previously published and validated with RV144 plasma samples (**Figure 1**) (29). The model predicts IgG-FcγR dimer complex formation (Ag : IgG:IgG : FcγR:FcγR) at steady state as a function of IgG subclass, antigen, and FcR dimer concentrations. In the model, two IgG antibodies bind each antigen before forming a complex with dimeric FcR. We obtained parameters for the model from literature and with measurements made previously (29) where median fluorescent intensity (MFI) of HIV env glycoprotein 120 (gp120) strain A244 (env) specific IgG1, IgG2, IgG3, and IgG4 was measured in the plasma of 105 RV144 vaccinees (8). We converted MFI measurements into a relative concentration measurement based on a reference concentration (17) of HIV-specific IgG in a similar vaccine trial. Though this reference concentration does not directly represent our plasma samples, we do not have the ability to directly measure concentration through the use of a standard curve, so the concentrations predicted throughout by the model are thus not to be used as absolute measures, but as relative measures.

### Evaluating Combined IgG1 Concentration and Affinity Parameter Changes

In order to evaluate the relative and combined roles of IgG1 allotype (i.e. IgG subclass changes) and FcR affinity (i.e. FcR polymorphisms), IgG1 affinity for FcγRIIIA-V^158^ or IgG1 concentration were held constant at its baseline value (listed in the parameter table in **Figure 1C**), while the other parameter was varied over 50 values spanning 1.7-256 nM or 2e-6-8e-4 nm^-1^s^-1^. Model outputs from all simulations were subtracted by the baseline complex formation to calculate the difference in complex formation for each condition. We simulated 2,500 different combinations of IgG1 concentration and IgG1 affinity for FcγRIIIA-V^158^ spanning 1.7-256 nM or 2e-6-8e-4 nm^-1^s^-1^ respectively while holding all other model parameters at baseline. We then subtracted each of these values by the model output with both IgG1 affinity and concentration at baseline. To identify regions where synergy between IgG1 concentration and IgG1 affinity for FcγR occurred, we used element-wise subtraction of the additive simulations (parameters were altered in isolation and added together) from simulations where parameters altered together in the model. The range of possible IgG1 concentration values was calculated by multiplying the maximum and minimum calculated IgG1 concentrations in the RV144 plasma samples (29) by each allotype conversion factor and taking the minimum and maximum results across all possible allotypes (23). Maximum and minimum IgG1 affinity values were selected as the highest and lowest affinity glycosylation forms of IgG1 across all FcgRIIIA polymorphisms (30).

### Evaluating Boosting of IgG1 Concentrations in Individuals With Different FcγRIIIa Polymorphisms

In order to model how changes in IgG subclass concentrations (that may occur upon vaccine boosting) can influence IgG-FcγR complex formation in individuals with different FcγRIIIa polymorphisms, we used the model to predict complex formation for each polymorphism by altering initial IgG1 and IgG3 concentrations from 0.004X to 20X baseline (post-vaccination measurements) in 2,500 different combinations. Affinity values for each FcγRIIIa polymorphism to each IgG subclass were used from previously published literature (11). We used IgG1 and IgG3 titers measured in RV144 vaccinees post-vaccination and after a simulated 170% IgG1 boost. This boosting value was chosen by using the highest fold change in HIV-specific Ab titers recorded in the RV306 follow up trial from 26 weeks (our initial post-vaccination timepoint) and after boosting in group 4b with AIDSVAX B/E and ALVAC-HIV at 18.5 months (31). A Wilcoxon matched pairs signed rank test was used to evaluate the difference in predicted complex formation for each individual across the two polymorphisms, both before and after boosting. All parameters besides initial IgG1 and IgG3 remained at their baseline value listed in the parameter table (**Figure 1C**) for all the above-described simulations. Specific IgG1 and IgG3 values were chosen using MATLAB’s log spacing function, logspace(), to give 50 values between 0.004X and 20X baseline.

### Simulating IgG1 Allotypes and Glycosylation

Baseline IgG subclass initial concentrations from all 105 RV144 vaccinees were assumed to be the G1m1,3 (15) IgG1 allotype. These were then converted into G1m1 and G1m1,3 for simulations based on conversion factors for initial IgG1, IgG2, IgG3 and IgG4 concentration as previously published (21), which were estimated using allotyped human plasma samples from previous a Phase I HIV vaccine trial (17). To predict affinity changes resulting from glycosylation, we estimated those that would be expected from afucosylation of IgG1 by taking the highest fold change for affinity of IgG1 to FcγRIIIa-V^158^ (31X; 62*10^-3^ nM^-1^s^-1^) reported in the literature (30). This high affinity glycosylation (afuscosylation with hyper-galactosylation and bisection) was compared to a baseline affinity (2*10^-3^ nM^-1^s^-1^).

In order to evaluate affinity changes resulting from glycosylation, projected upon all vaccinees for each of the three allotypes, the IgG-FcR immune complex formation was simulated at baseline, and the difference between each individual’s complex formation at baseline and with glycosylation for each allotyped population and compared them with a Friedman test with Dunn’s multiple comparisons in GraphPad Prism.

Allotype projections were performed as previously published (29), by first calculating the conversion factor. Under the assumption that the original RV144 data was G1m1,3 (15), the conversion factor was calculated to generate the corresponding IgG subclass concentrations for the G1m-1,3 and G1m1 allotypes. To calculate this value, we found the mean concentration for each IgG within each allotype from human plasma samples analyzed in Kratochvil et al. (17). Then, these values were divided by the corresponding mean IgG concentration for samples with the G1m1,3 allotype.


$$cf_{G1mj}^{IgGi}=conversion\;factor\;for\;IgGi\;to\;allotype\;G1mj$$

$$m_{G1mj}^{IgGi}=mean\;concentration\;of\;IgGi\;in\;allotype\;G1mj$$

$$cf_{G1mj}^{IgGi}=m_{G1mj}^{IgGi}/m_{G1m1,3}^{IgGi}$$

Each vaccinee’s initial IgG concentrations and baseline initial IgG concentrations were converted using the respective conversion factors as follows:


$$IgGi_{G1mj}^{x}=Initial\;IgGi\;concentration\;for\;vaccinee\;x\;in\;allotype\;G1mj$$

$$IgGi_{G1mj}^{x}=cf_{G1mj}^{IgGi}*IgGi_{G1m1,3}^{x}$$

### Determining Preferred Boosting Method in IgG1 Allotypes

Simulations as above, projecting all 105 RV144 vaccinees as the three IgG1 allotypes and two FcRIIIA polymorphisms (FcγRIIIa-V^158^ and FcγRIIIa-F^158^) were calculated, providing predictions for six different genetic combinations (**Figure 5A**). In each of these six genotypes we then simulated a boost in either IgG1 initial concentration or k_on_ IgG1-FcR (ie IgG1 affinity to FcR) by 10%, 25%, 50%, 75%, 100%, 250%, 500%, 750%, or 1000% above their personal baseline. The six genotypes were compared at baseline using a Friedman test with Dunn’s multiple comparisons in GraphPad Prism 9.

To modify the original parameter to include the boost, a new concentration or affinity was calculated using the following formula, where the original parameter is specific to the individual and genotype:


New parameter=original parameter+(original parameter∗boost)

### Evaluating Mixed Allotype Populations

To determine the importance of affinity and concentration-based interventions within 10 mixed allotype populations, simulations were run as described above projecting all 105 RV144 vaccinees into different allotypes. Within this analysis, 10 mixed allotype populations were simulated with varying proportions of individuals assigned to each allotype. Each allotype is represented in each population at 100%, 66%, 33%, 17%, or 0% (see **Figure 6** for specific breakdowns). Each vaccinee (n = 105) was randomly assigned an allotype to fulfill the population breakdown. In populations where vaccinees couldn’t be evenly split into the population’s allotypes, remaining vaccinees were again randomly assigned an allotype (i.e. 70 vaccinees assigned to G1m1, 18 assigned to G1m1,3 and 17 to G1m-1,3 in Population F). We performed this randomized vaccinee allotype assignment 25 times for each population to create a more robust and representative population n = 2,625 for each population. All simulations were run with FcγRIIIa-V^158^ affinity values.

## Results

### Synergism Between IgG1 Concentration and IgG1 Affinity

**Figure 2:**
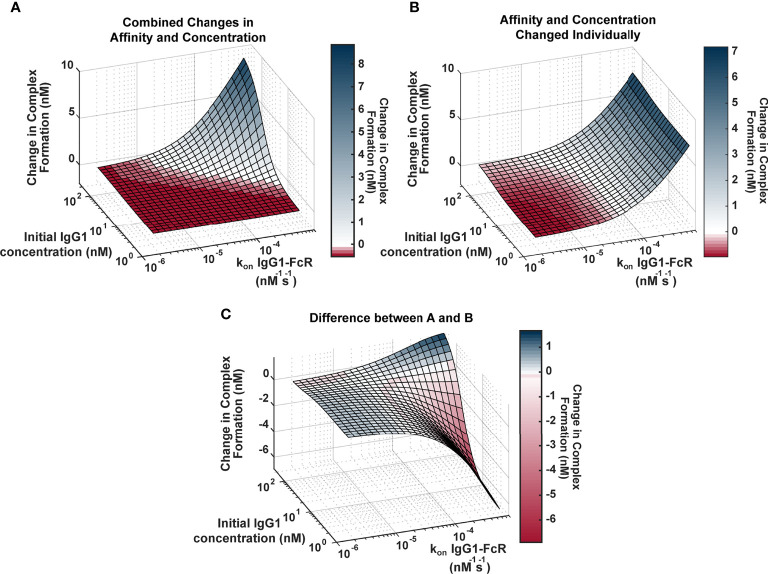
Landscape illustrating the relationships between IgG1 concentration and IgG1- FcγR affinity across the physiological landscape of parameters (2500 unique parameter combinations). **(A)** Model predictions for the change in complex formation from baseline when IgG1 initial concentration (x axis) and k_on_ IgG1- FcγR (y axis) were altered individually and the resulting change in complex formation is added together (z axis). Color indicates predicted change in complex formation from baseline. **(B)** Model predictions for the change in complex formation from baseline when both parameters are altered simultaneously in the model. Color indicates predicted change in complex formation from baseline. **(C)** The difference between **(A, B)**, illustrating parameter combinations where synergy occurs. Blue indicates positive synergy, where the combined parameter changes **(B)** result in greater complex formation compared to was predicted by separate changes added together **(A)**, white indicates no synergy, and red indicates anergy; where the combined parameter changes **(B)** result in lower complex formation compared to was predicted by separate changes added together **(A)**.

Genetic background has the potential to influence both IgG1 concentration (via IgG1 allotypes) and IgG1 binding affinity for FcR (via FcR polymorphisms). In order to better understand the relationship between these two parameters and how they influence FcγRIIIa activation, we applied an ODE model to predict Antigen-IgG-FcR immune complex formation as both parameters were altered simultaneously over a physiological range of 2500 unique parameter combinations (**Figure 2A**). The resulting landscape illustrated the interdependence of these two parameters, and how simultaneous changes have the potential for a synergistic influence on complex formation. Specifically, IgG1 affinity was only effective for increasing complex formation, upon IgG1 titers surpassing specific concentration thresholds (around ~10-230 nM depending on the affinity value). Likewise, increasing IgG1 concentration had a limited effect, which was determined by IgG1 affinity. Furthermore, in situations where both IgG1 concentration and IgG1 affinity were high (~200 nM and ~7e-4 nM^-1^s^-1^, respectively), the model predicted non-linear increases in complex formation, beyond what would be predicted from adding the changes resulting from both parameters individually.

To illustrate the synergistic result of modulating multiple parameters more clearly, we created a second surface that predicted complex formation, if IgG1 concentration and IgG1 affinity were altered separately in the model and resulting changes were added together (**Figure 2B**). The surface represents what would be expected if changes in IgG1 concentration and affinity were considered separately in isolation, and notable features include: 1) the ability of each parameter to influence complex formation without the other; and 2) absence of the potential for very high complex formation when both parameters are high.

To identify specific parameter ranges where synergisms or anergisms occur (combined changes are greater than or less than what would be expected from separate parameter changes added together), we next subtracted “additive” (parameters changed separately; **Figure 2B**) surface from the “combined” surface (parameters changed simultaneously; **Figure 2A**) to create **Figure 2C**. Positive regions of this surface (blue) indicate regions where combined parameters changes are much greater than what would be expected from adding separate changes, whereas the negative regions (red) represent parameter combinations where actual changes would be much less than what would be expected from adding individual changes. This landscape indicates the potential for synergistic complex formation (blue) when both concentration and affinity are high (102-230 nM, and 2.9e-5-7e-4 nM^-1^s^-1^). Interestingly it also illustrates the potential to overestimate complex formation when IgG1 affinity is high, but IgG1 concentration is low (1.7-102 nM, and 2.9e-5-7e-4 nM^-1^s^-1^). Altogether these results have important implications for how genetic background (which has the capacity to alter both IgG1 concentration and IgG1 affinity for FcγR) may influence FcγR activation after vaccination and may allow for more rational design of vaccine interventions.

### FcR Polymorphism Influences FcγR Activation After Boosting

One interesting result of the previous simulations in **Figure 2** was that there is a limit in the effects of increasing IgG1 concentration alone, and at higher IgG1 concentrations, IgG1 affinity determines the limit. This result has implications for vaccine boosting in individuals with different FcR polymorphisms. We hypothesized that the effect of boosting (large changes in IgG1 concentration) would be limited in individuals with the low affinity FcγRIIIa-F^158^ polymorphism, whereas it would be much higher in individuals with the higher affinity FcγRIIIa-V^158^ polymorphism. Therefore we hypothesized that the differences in immune complex formation between the two polymorphisms would become even greater after boosting (compared to first vaccination).

**Figure 3:**
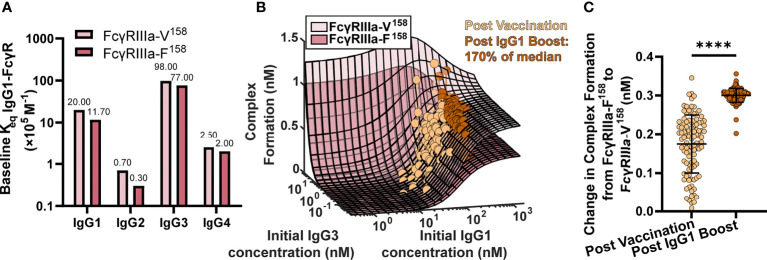
FcγR polymorphisms have a greater influence on complex formation after IgG1 boosting. **(A)** Baseline K_eq_ of each IgG subtype to the high affinity FcγRIIIa-V^158^ polymorphism (light pink) and the low affinity FcγRIIIa-F^158^ polymorphism (dark pink) as reported by Bruhns et al. (11). **(B)** Complex formation (z axis) predicted by the model for 2500 combinations of initial IgG1 and IgG3 concentration (x and y axes) for FcγRIIIa-V^158^ (light pink) and FcγRIIIa-F^158^ (dark pink). Each dot represents an RV144 plasma sample (n=105) with respective initial IgG1 and IgG3 concentrations plotted post-vaccination (baseline-light orange), and after a simulated 170% (145 nm) boost of IgG1 (dark orange). The simulated boost magnitude was estimated based on the highest fold change seen in RV306 between 26 weeks and peak HIV specific IgG titer (2.64X in arm 4b) (31). **(C)** The difference in complex formation predicted between the FcγRIIIa-F^158^ and FcγRIIIa-V^158^ polymorphisms post-vaccination (light orange) and post-IgG1 boost (dark orange; Wilcoxon matched-pairs signed rank test; ****p-value < 0.0001).

To test this idea, we ran simulations for the high and low affinity FcγRIIIa polymorphisms by changing the affinity for all IgGs to FcγRIIIa according to published values (11) (FcγRIIIa-V^158^ light pink, and FcγRIIIa-F^158^ dark pink, respectively; **Figure 3A**) at 2,500 different initial IgG1 and IgG3 concentration combinations with all other parameters maintained using baseline values (FcγRIIIa-V^158^ light pink, and FcγRIIIa-F^158^ dark pink; **Figure 3B**). IgG1 and IgG3 have previously been identified as the significant IgG subtypes of importance (29) due to IgG1’s high initial concentration and IgG3’s high affinity to FcR (**Figures 1C**, **3A**). The resulting profile of both polymorphism surfaces revealed that changes in IgG1 concentration were predicted to increase complex formation up to a certain point, illustrated by a plateau around 300 nM, after which no additional changes in complex formation would be predicted regardless of IgG1 increases. Comparing results for the two polymorphisms (light pink vs. dark pink surface) revealed that the biggest differences between polymorphisms occur in the plateaus regions, when IgG1 concentration is high; specifically, the FcγRIIIa-V^158^ polymorphism plateau is 66% higher than the FcγRIIIa-F^158^ plateau.

Based on individual IgG1 and IgG3 initial concentrations measured in the RV144 plasma samples (n=105) we plotted each individual on both surfaces at baseline (light orange), and after a simulated boost (31) in IgG1 concentration (dark orange; **Figure 3B**). After first vaccination, many vaccinees were predicted to be in an IgG1 sensitive region, regardless of FcR polymorphism. However, an increase in antigen-specific IgG1 (similar to the boost applied in RV306) moves many vaccinees from the IgG1 sensitive region (30-300 nM) onto or nearing the plateau region, where complex formation is highly dependent on FcR polymorphism. Indeed, the difference in complex formation between the polymorphisms after boosting was significantly greater than it was at baseline (after first vaccination) (Wilcoxon matched-pair rank test, p < 0.0001; **Figure 3C**).

### The G1m-1,3 IgG1 Allotype Is Not Predicted to be Sensitive to IgG1 Fc Glycosylation Modifications

Model results in **Figure 2** revealed the potential for unexpected interactions between IgG1 concentration and IgG1 affinity. In a setting with low IgG1 concentration, there is the potential that large increases in IgG1 affinity to FcγR will have little to no effect on IgG-FcγR complex formation. Conversely, at high IgG1 concentrations, results revealed the potential for non-linear increases in complex formation. Based on these observations, we used the model to assess how IgG1 concentration differences in IgG1 allotypes may influence sensitivity to FcR affinity modifications (e.g. glycosylation).

**Figure 4:**
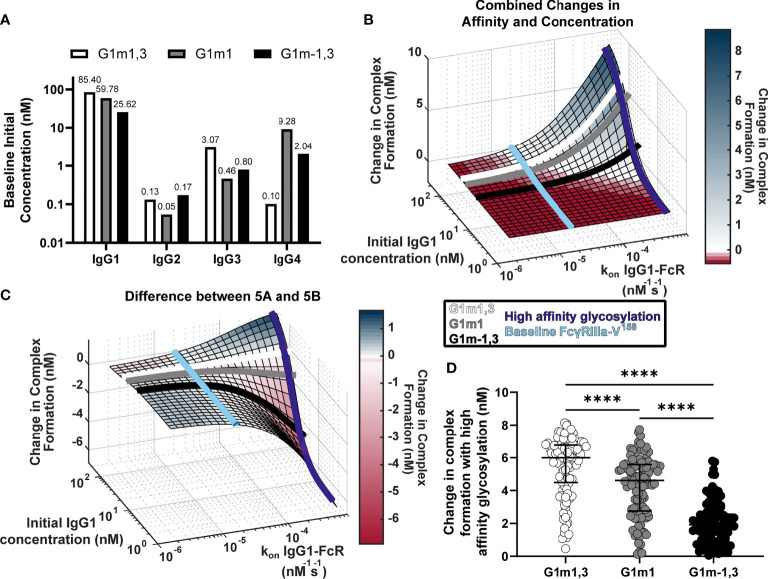
Glycosylation differentially impacts IgG1 allotypes. **(A)** Expected IgG1, IgG2, IgG3, and IgG4 concentrations for G1m1,3 (white), G1m1 (gray), and G1m-1,3 (black) allotypes based on previously published work (17, 29). **(B)** Model predictions for complex formation as IgG1 concentration and k_on_ IgG1- FcγR are altered over physiological ranges (**Figure 2B**). Lines indicate IgG1 concentrations for three different IgG1 allotypes (G1m1,3 (white), G1m1 (gray), G1m-1,3 (black)), and the affinity change expected from an afucosylation glycosylation modification (purple) compared to baseline (light blue). **(C)** The difference (**Figure 2C**) between the combined parameter change surface (**Figure 2A**) and the additive surface (**Figure 2B**). Lines indicate IgG1 concentrations for three different IgG1 allotypes (G1m1,3 (white), G1m1 (gray), G1m-1,3 (black)), and the affinity change expected from an afucosylation glycosylation modification (dark blue) compared to baseline FcgRIIIaV158 (light blue). **(D)** Change in complex formation from baseline affinity to an afucosylated affinity in each allotype, G1m1,3 (white), G1m1 (gray), and G1m-1,3 (black) (Friedman test with Dunn’s multiple comparisons test; ****p-value < 0.001).

Previous studies suggest that IgG1 allotype alters all four IgG subclass concentrations, hence we used these measurements to estimate the median IgG1, IgG2, IgG3 and IgG4 concentrations for each allotype (**Figure 4A**) (17). As the G1m1,3 allotype is expected to be prevalent in the original RV144 (Thai) population, we assumed all original RV144 vaccinees (n=105) were of the G1m1,3 (**Figure 4A**, white bar) allotype (25), which is expected to have higher IgG1 and IgG3 concentrations, compared to G1m1 (gray bar) and G1m-1,3 (black bar) which have higher IgG4.

Using results in **Figure 2**, we plotted each IgG1 allotype on the surface based on expected median IgG1 concentration (**Figures 4B, C**). Using this same principle, we also added lines showing where the baseline affinity measurement is for FcγRIIIa-V^158^ (light blue, 2e-5 nM^-1^s^-1^) as well as potential maximal increases in affinity similar to what would be expected with an IgG1 Fc afucosylation modification (purple, 62e-5 nM^-1^s^-1^) based on values in the published literature (30). Results indicate that G1m1,3 and G1m1 allotypes are expected to follow similar trajectories, where increases in affinity would considerably increase complex formation after ~3e-5 nM^-1^s^-1^ reaching complex formation levels of 6.5 nM and 5.2 nM respectively. Conversely for the G1m-1,3 allotype (lower IgG1 concentration) the model illustrates how similar glycosylation modification would result in much lower complex formation [only ~1.8 nM complex formation after a high affinity glycosylation modification (**Figure 4B**)].

Plotting the same lines representing IgG1 allotypes and FcRs onto a second surface illustrating the differences between combined changes in concentration and affinity and the individually changed analysis, we see that at baseline FcγRIIIa-V^158^ affinity values (**Figure 4C**, light blue) the predicted combined effects of IgG concentration changes are not much different between an individual and additive method. In contrast, after afucosylation, the additive method would overestimate complex formation in G1m-1,3 by 4.3 nM, while it is only slightly different in G1m1 (1.1 nM) and G1m1,3 (0.08 nM) (**Figure 4C**). Using the same conversion factors as above, we projected every RV144 vaccinee from G1m1,3 into G1m1 and G1m-1,3, and simulated each individual’s complex formation after RV144 first vaccination and with the afucosylation change in affinity. Unsurprisingly, the change in complex formation with afucosylation was significantly different in each allotype following the trend of median IgG1 concentration (Median change in complex formation: G1m1,3, 6.0 nM; G1m1 4.6 nM; G1m-1,3 1.9 nM; Friedman test with Dunn’s multiple comparisons, all p<0.0001) (**Figure 4D**).

### IgG1 Allotype Determines Whether Vaccine Boosts That Increase IgG1 Concentration vs. Boosts That Increase IgG1 Affinity Would Be More Effective for Improving FcR Activation

**Figure 5:**
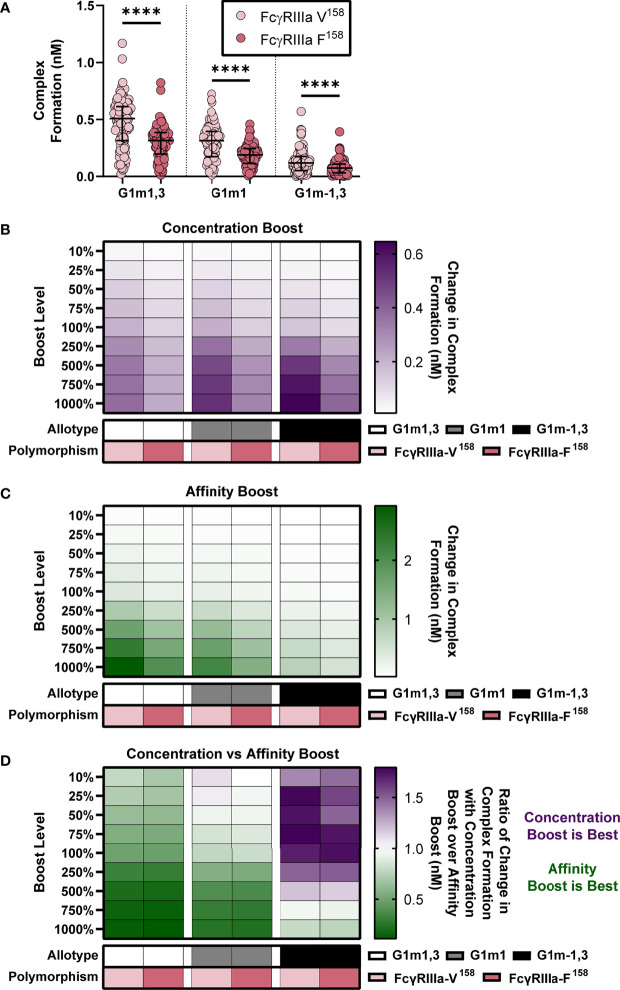
IgG1 allotype determines whether boosting IgG1 concentration or boosting IgG1 affinity (k_on_ IgG1- FcγR) would be most effective for increasing complex formation. **(A)** Model predictions for complex formation of RV144 vaccinees (n=105) in two FcγRIIIa polymorphisms, FcγRIIIa-V^158^ (light pink) and FcγRIIIa-F^158^ (dark pink), and three IgG1 allotypes, G1m1,3 (original RV144 data), G1m1 and G1m-1,3. Polymorphisms were simulated by altering the binding affinities of each IgG subtype to FcγR as previously published (11) and indicated in **Figure 3A**. Allotypes are simulated by multiplying each vaccinee’s IgG1, IgG2, IgG3 and IgG4 initial concentration by its respective conversion factor as previously published (29) and indicated in **Figure 4A** (Friedman test with Dunn’s multiple comparisons test comparing the two polymorphisms within each allotype; ****p-value < 0.001). **(B)** Simulated IgG1 concentration boosting in each allotype (G1m1,3, white; G1m1, gray; G1m-1,3 black) and polymorphism (FcγRIIIa-V^158^, light pink; FcγRIIIa-F^158^, dark pink) combination. Boosts were calculated by multiplying the individual’s baseline initial IgG1 concentration value by the boost levels and then this was added on top of each individual’s baseline. **(B)** Color indicates median change in complex formation for each genetic background. **(C)** Simulated boosting of k_on_ IgG1- FcγR in each allotype (G1m1,3, white; G1m1, gray; G1m-1,3 black) and polymorphism (FcγRIIIa-V^158^, light pink; FcγRIIIa-F^158^, dark pink) combination. Boosts were calculated by multiplying the individual’s baseline k_on_ IgG1- FcγR value by the boost levels and then this was added on top of each individual’s baseline. Color indicates median change in complex formation for each genetic background and boost as indicated. **(D)** The ratio of median change in complex formation with a boost in IgG1 concentration over median change in complex formation with a boost in k_on_ IgG1-FcγR (affinity) at each boosting level. This ratio shows which type of boost is most effective for increasing complex formation (IgG1 concentration, purple; k_on_ IgG1-FcγR, green) and when both are equally beneficial (white).

Our model results suggest that the effect of changes in IgG1 concentration varies depending on a given IgG1 affinity to FcR. One intriguing implication of this result is that individuals with different IgG1 allotypes (different baseline IgG1 concentration) could be differentially sensitive to vaccines that increase antibody titers (IgG1 concentration) vs. adjuvants that modify IgG1 affinity *via* glycosylation. To explore this idea quantitatively, we simulated 6 different genotypes (FcγRIIIa-F^158^ and FcγRIIIa-V^158^ polymorphisms in the G1m1,3, G1m1 and G1m-1,3 allotypes). As expected we found significant differences in complex formation across all 6 genotypes (**Figure 5A**).

We then simulated nine different boosts, 10%-1000% above values after first vaccination for either IgG1 concentration (**Figure 5B**) or IgG1 affinity (**Figure 5C**) in all vaccinees. We used the median change in complex formation for each genetic background and boosting level to create heatmaps that illustrate the expected resulting change in complex formation. Intriguingly, results illustrated how concentration boosting (increasing antibody titers) has a larger effect on the allotypes with lower initial IgG1 concentration (**Figure 5B**) and that affinity boosts have a larger effect on the allotypes with higher initial IgG1 concentration (**Figure 5C**).

In order to definitively show which type of boosting is optimal for each boosting level and genetic background, we calculated the ratio of change in complex formation with a boost in IgG1 concentration over change in complex formation with a boost in IgG1 affinity to FcγRIIIa (**Figure 5D**). The resulting heat maps illustrates how concentration boosting is predicted to be more beneficial than affinity boosting for the G1m-1,3 allotype until 750% (purple). The lower starting concentration of IgG1 in G1m-1,3 (median IgG1 25.62 nM) prevents affinity changes from improving complex formation until it reaches at least 1e-4 nM^-1^s^-1^. Conversely, model results indicated that the G1m1,3 and G1m1 allotypes (with higher starting IgG1 concentrations) would be most responsive to changes in affinity (**Figure 5D**). Overall, these results suggest specific vaccine interventions that may be differentially effective for inducing improved Fc effector functions for individuals with different IgG1 allotypes. A separate analysis of FcγRIIa resulted in a similar outcome (**Figure S1**).

### Amount of G1m-1,3 Allotype in a Population Determines Whether Boosting IgG1 Antibody Titers Will Be Effective

**Figure 6:**
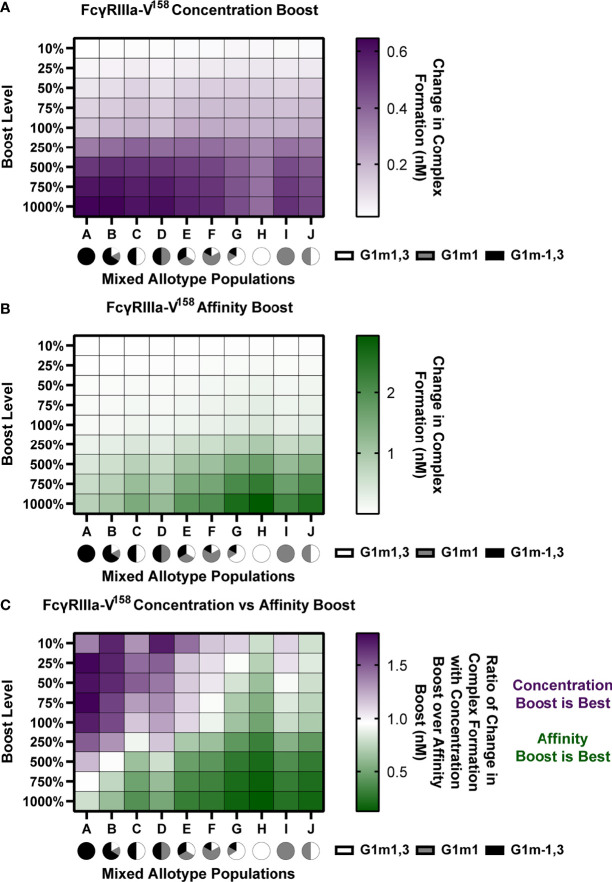
In mixed allotype populations, the benefit of boosting IgG1 concentration vs. IgG1 affinity is dependent on the presence of the G1m-1,3 allotype. **(A)** Boosting of initial IgG1 concentration in mixed allotype populations (G1m1,3, white; G1m1, gray; G1m-1,3 black) for FcγRIIIa-V^158^. Color indicates predicted change in complex formation **(B)** Boosting of k_on_ IgG1- FcγR in mixed allotype populations (G1m1,3, white; G1m1, gray; G1m-1,3 black). Color indicates predicted change in complex formation **(C)** The ratio of median change in complex formation with a boost in IgG1 over median change in complex formation with a boost in k_on_ IgG1-FcγR at each boosting level. This ratio indicates which type of boost is predicted to be most effective for increasing complex formation (IgG1 concentration, purple; k_on_ IgG1-FcγR, green).

Given that the model predicts that IgG1 allotype drives the preferred boosting type and that many populations worldwide have different allotype distributions, we next simulated boosting in mixed allotype populations with FcγRIIIa-V^158^. These populations were simulated by randomly assigning vaccinees to an allotype based on the given ratio of allotypes for the indicated population (Populations A-J; **Figure 6**). Each individual was then projected into their assigned allotype. To be robust in these assignments, this was repeated 25 times for each population and the data was pooled (n = 2,625 for each population).

We performed both IgG1 concentration and IgG1 affinity boosting as described above (**Figure 6**). Overall, we found that the populations with majority G1m-1,3 (populations A-D) benefit more from concentration boosts, and populations higher in G1m1,3 benefit more from FcR affinity boosts (populations G, H, and J) (**Figures 6A, B**). Interestingly, population C, which was 50% G1m1,3, and 50% G1m-1,3, only gained minimal benefits from affinity boosts compared to populations G, H and J, (**Figure 6B**). When we evaluated the ratio of change in complex formation from a concentration boost over change with an affinity boost, we found IgG1 concentration boosting to be beneficial for almost all populations at the lowest boosting level (10-25%), but only remained beneficial at higher boosting levels in populations with a higher prevalence of G1m-1,3 allotypes (**Figure 6C**). Notably the level at which affinity boosting becomes more beneficial than concentration boosting seems to closely follow the level of G1m-1,3 within the population and this holds true for FcγRIIIa-V^158^, FcγRIIIa-H^131^, and FcγRIIIa-R^131^ (**Figures S2**–**S4**). Altogether this suggests specific guidelines for rational vaccine design to improve FcγRIIIa activation in future trials with mixed allotype populations.

## Discussion

Here we identify specific mechanisms by which heterogeneity in FcγR activation after vaccination may be linked to IgG1 allotypes and FcγR polymorphisms. Importantly, we found that vaccine boosting regimens which increase IgG1 antibody titers may have limited utility in some allotypes (G1m1,3 and G1m1) and may be more effective in others (G1m-1,3). Instead, for G1m1,3 and G1m1 allotypes, vaccine boosting strategies that modulate IgG1 affinity to FcγR (e.g. *via* adjuvants that modify glycosylation) may be required to improve FcγR activation. The model also illustrates how the influence of FcγRIIIa affinity from different FcR polymorphisms is predicted to have limited influence upon FcR activation until higher IgG1 antibody titers are reached, such as those expected after vaccine boosting. These differences arise from synergistic relationships between IgG1 concentration and affinity for FcγR that could not have been predicted without a computational model.

The computational model also demonstrates how concurrent changes in antigen specific IgG1 antibody titers and IgG1 affinity for FcγR may have more (synergistic), or less (anergistic) of an effect on FcγR activation than previously appreciated. These results suggest that focusing vaccine design on either concentration or affinity alone may not have the expected result. The model identified specific values for IgG1 affinity to FcγR (~10^-4^ nM^-1^s^-1^ at baseline IgG1 concentration), that would need to be reached before changes IgG1 concentration will have a great effect (**Figure 2**). This can be visualized in **Figure 2C** where predictions of the additive effects of changes in affinity and concentration in isolation were often overestimated than the actual effects when both were changed in combination.

Perhaps one of the most important outcomes reported here is the potential for differential sensitivity of IgG1 allotypes to boosting regimens that increase antibody titers vs. vaccine adjuvants that may influence glycosylation profiles (i.e. FcR affinity). The model predicts that 2 of the 3 allotypes we evaluated would not be sensitive to boosting regimens that increased IgG1 concentration. This has implications for RV144 and associated follow-up trials, where different allotype distributions would be expected depending on geographic location. Though IgG1 allotype was not measured directly in RV144, the Thai population would likely have a greater prevalence of the G1m1,3 allotype compared to other trials conducted in South Africa, which have previously been reported to have greater prevalence of G1m1 and G1m-1,3 (32). Model results suggest that while an initial vaccination would be most effective in G1m1,3 (due to high baseline IgG1 titers), boosting regimens to increase IgG1 concentration may not improve Fc- mediated functions. Indeed RV305 (33) and RV306 (34) conducted in Thai populations did increase HIV-specific IgG titers, but to our knowledge the resulting changes in FcγR activation have not yet been evaluated. While the model suggests that FcγR polymorphism is not essential in determining which boosting type and boosting level will be most beneficial (**Figure 6**), it would still make an impact in individuals with relatively high HIV specific IgG1 titers (G1m1,3 and G1m1).

A key limitation is the study is the evaluation of only one FcγR type (FcγRIIIa) and one binding site on one antigen, though we would expect similar results for different FcγRs and antigen binding sites (29). Future models could be expanded to examine multiple FcRs simultaneously in the case of individuals heterozygous for FcR polymorphism or to investigate FcR type competition. Furthermore, this study is based only upon assumed IgG1 allotypic distributions. Though IgG1 allotype measurements would be ideal for validating model findings, they were not available for the samples used in this analysis. Future experimental vaccine studies using samples with known allotype and FcR polymorphism information will be needed to be conducted to confirm this study.

Overall, this study illustrates several different scenarios where host genetics is predicted to influence Fc effector responses upon vaccine boosting and that different vaccine boosting regimens are likely to have varied benefits depending on host genotypes. Specifically the model could use genetic background to guide the focus of vaccine regimens towards concentration boosting or adjuvant adjustments that affect affinity values. Given that Fc effector functions have been demonstrated to be important for the control and protection of numerous other infectious diseases including COVID-19 and influenza where vaccine boosting regimens are currently being implemented (1, 35–37), future studies that explore the influence of antibody allotypes and FcR polymorphism upon these vaccine boosting strategies could provide valuable insight.

## Data Availability Statement

The original contributions presented in this study are included in the article/**Supplementary Material**. The code for this study can be found on GitHub at https://github.com/melissalemke/FcR-ODE-Genetics. Further inquiries can be directed to the corresponding authors.

## Ethics Statement

Experimental measurements used here were part of previously published study that was reviewed and approved by the Institutional Review Board at the University of Melbourne. All data provided for this study was de-identified of demographics (including gender and age). The study was reviewed by the Institutional Review Board at the University of Michigan and determined to be “Not Regulated” (HUM00191689).

## Author Contributions

ML designed the study, performed computational analysis, created figures, and wrote the manuscript. RT and EB performed computational analysis and edited the manuscript. MM and EL performed experimental measurements and edited the manuscript. SJK provided information used to calculate conversion factors for IgG1 allotypes and edited the manuscript. SR-N, PP, and SN conducted the RV144 Vaccine trial. BW and PH provided rsFcγR dimers and edited the manuscript. SJK arranged institutional ethics and edited the manuscript. AC designed the study, wrote the manuscript, and oversaw experimental analysis. KA designed the study, wrote the manuscript, and oversaw computational analysis. All authors contributed to the article and approved the submitted version.

## Funding

This work was supported by the Australia National Health & Medical Research Center (NHMRC) (APP1125164 to AC and GNT1145303 to PMH and BW) and the American Foundation for AIDS Research (amfAR) Mathilde Krim Fellowship (109499-61-RKVA) to AC, and by start-up funds from the University of Michigan to KA. SJK and AC are supported by NHMRC fellowships.

## Conflict of Interest

The authors declare that the research was conducted in the absence of any commercial or financial relationships that could be construed as a potential conflict of interest.

## Publisher’s Note

All claims expressed in this article are solely those of the authors and do not necessarily represent those of their affiliated organizations, or those of the publisher, the editors and the reviewers. Any product that may be evaluated in this article, or claim that may be made by its manufacturer, is not guaranteed or endorsed by the publisher.
