# Supplementary material for: A Quantitative Approach to Unravel the Role of Host Genetics in IgG-FcγR Complex Formation After Vaccination
Source: Front Immunol. 2022 Feb 22;13:820148. doi: 10.3389/fimmu.2022.820148 (PMC8902241; doi:10.3389/fimmu.2022.820148)
Supplement: Supplementary file 1 [file DataSheet_1.docx]

Supplementary Material

# Supplementary Figures


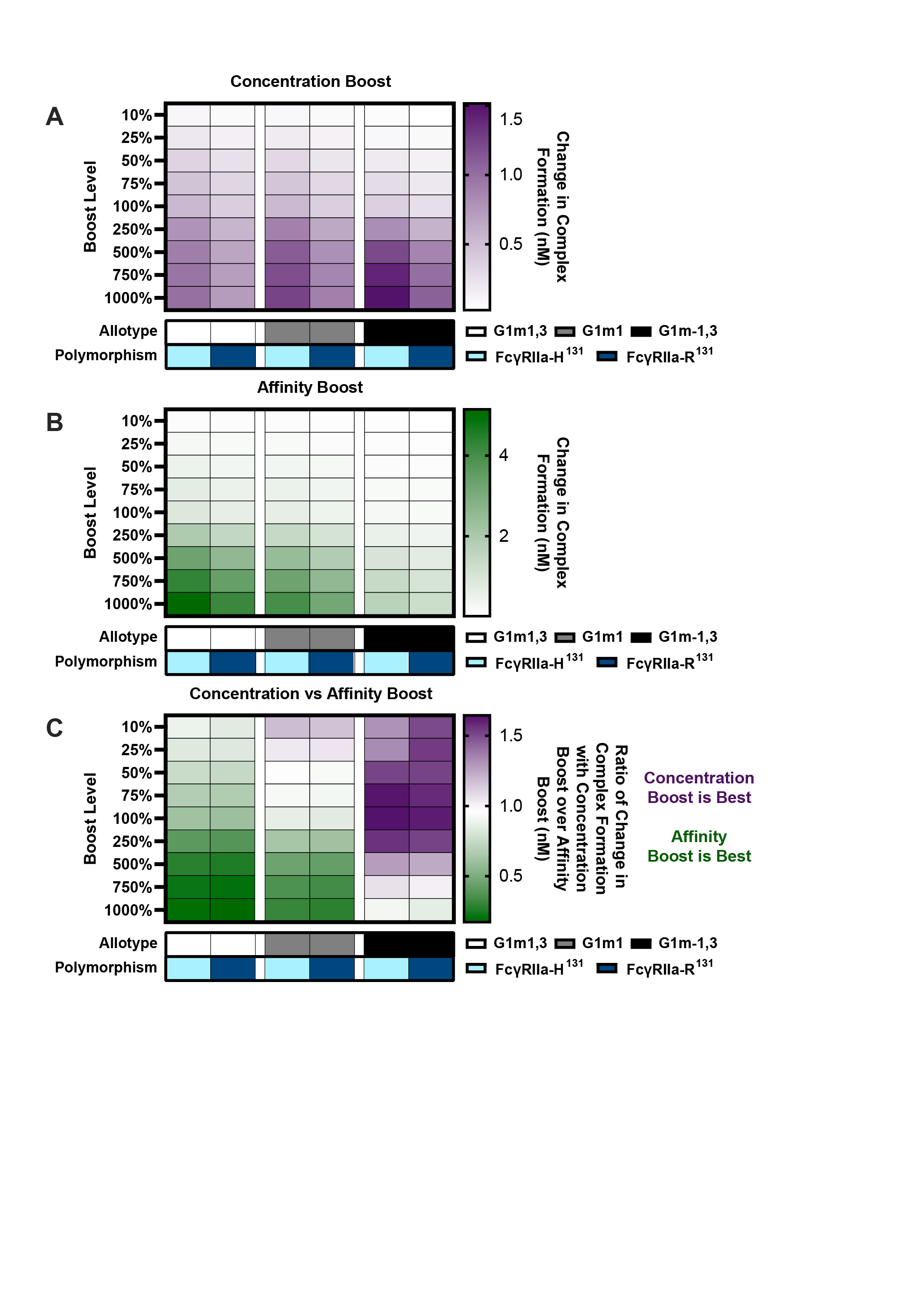
 **Supplementary Figure 1.** **IgG1 allotype determines whether boosting IgG1 concentration or boosting IgG1 affinity (k_on_ IgG1- FcγR) would be most effective for increasing complex formation in FcRIIa as well.** (A) Simulated IgG1 concentration boosting in each allotype (G1m1,3, white; G1m1, gray; G1m-1,3 black) and polymorphism (FcγRIIa-H^131^, light blue; FcγRIIa-R^131^, dark blue) combination. Boosts were calculated by multiplying the individual’s baseline initial IgG1 concentration value by the boost levels and then this was added on top of each individual’s baseline. Color indicates median change in complex formation for each genetic background. (B) Simulated boosting of k_on_ IgG1- FcγR in each allotype (G1m1,3, white; G1m1, gray; G1m-1,3 black) and polymorphism (FcγRIIa-H^131^, light blue; FcγRIIa-R^131^, dark blue) combination. Boosts were calculated by multiplying the individual’s baseline k_on_ IgG1- FcγR value by the boost levels and then this was added on top of each individual’s baseline. Color indicates median change in complex formation for each genetic background and boost as indicated. (C) The ratio of median change in complex formation with a boost in IgG1 concentration over median change in complex formation with a boost in k_on_ IgG1-FcγR (affinity) at each boosting level. This ratio shows which type of boost is most effective for increasing complex formation (IgG1 concentration, purple; k_on_ IgG1-FcγR, green) and when both are equally beneficial (white).


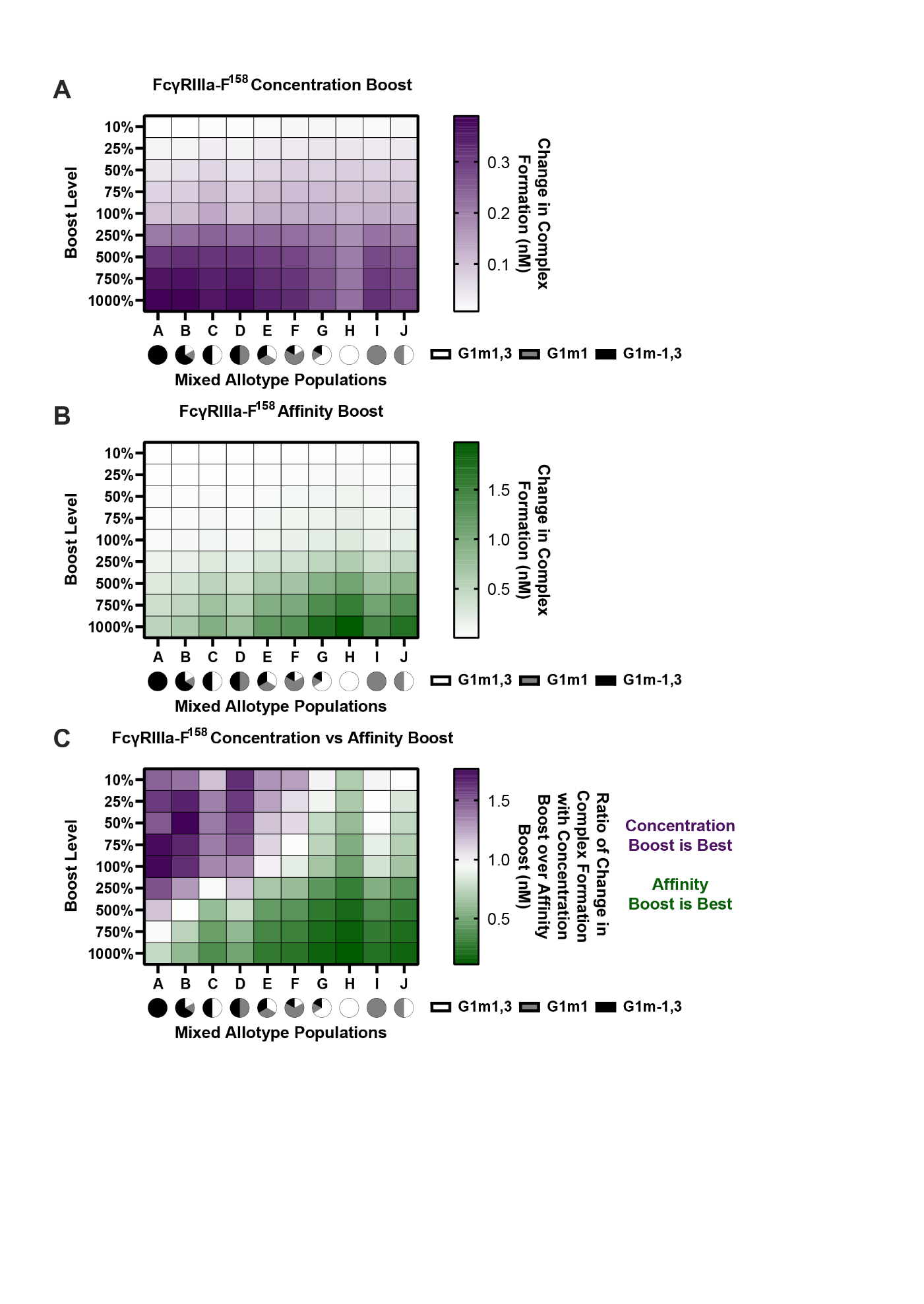
 **Supplementary Figure 2.** **In mixed allotype populations with FcRIIIa-F^158^, the benefit of boosting IgG1 concentration vs. IgG1 affinity is dependent on the presence of the G1m-1,3 allotype.** (A) Boosting of initial IgG1 concentration in mixed allotype populations (G1m1,3, white; G1m1, gray; G1m-1,3 black) for FcγRIIIa-F^158^. Color indicates predicted change in complex formation (B) Boosting of k_on_ IgG1- FcγR in mixed allotype populations (G1m1,3, white; G1m1, gray; G1m-1,3 black). Color indicates predicted change in complex formation (C) The ratio of median change in complex formation with a boost in IgG1 over median change in complex formation with a boost in k_on_ IgG1-FcγR at each boosting level. This ratio indicates which type of boost is predicted to be most effective for increasing complex formation (IgG1 concentration, purple; k_on_ IgG1-FcγR, green).


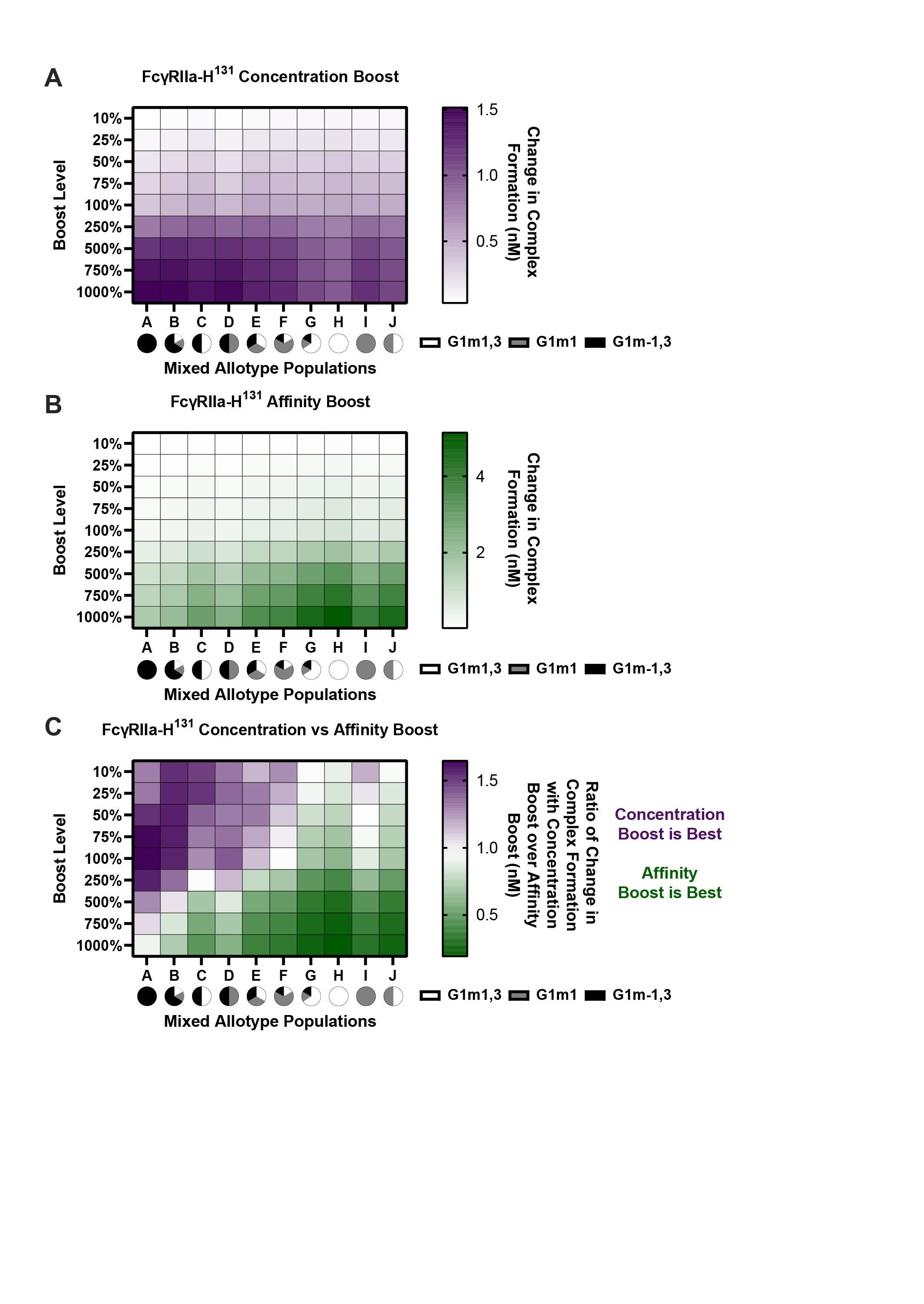
 **Supplementary Figure 3.** **In mixed allotype populations with FcRIIa-H^131^, the benefit of boosting IgG1 concentration vs. IgG1 affinity is dependent on the presence of the G1m-1,3 allotype.** (A) Boosting of initial IgG1 concentration in mixed allotype populations (G1m1,3, white; G1m1, gray; G1m-1,3 black) for FcγRIIa-H^131^. Color indicates predicted change in complex formation (B) Boosting of k_on_ IgG1- FcγR in mixed allotype populations (G1m1,3, white; G1m1, gray; G1m-1,3 black). Color indicates predicted change in complex formation (C) The ratio of median change in complex formation with a boost in IgG1 over median change in complex formation with a boost in k_on_ IgG1-FcγR at each boosting level. This ratio indicates which type of boost is predicted to be most effective for increasing complex formation (IgG1 concentration, purple; k_on_
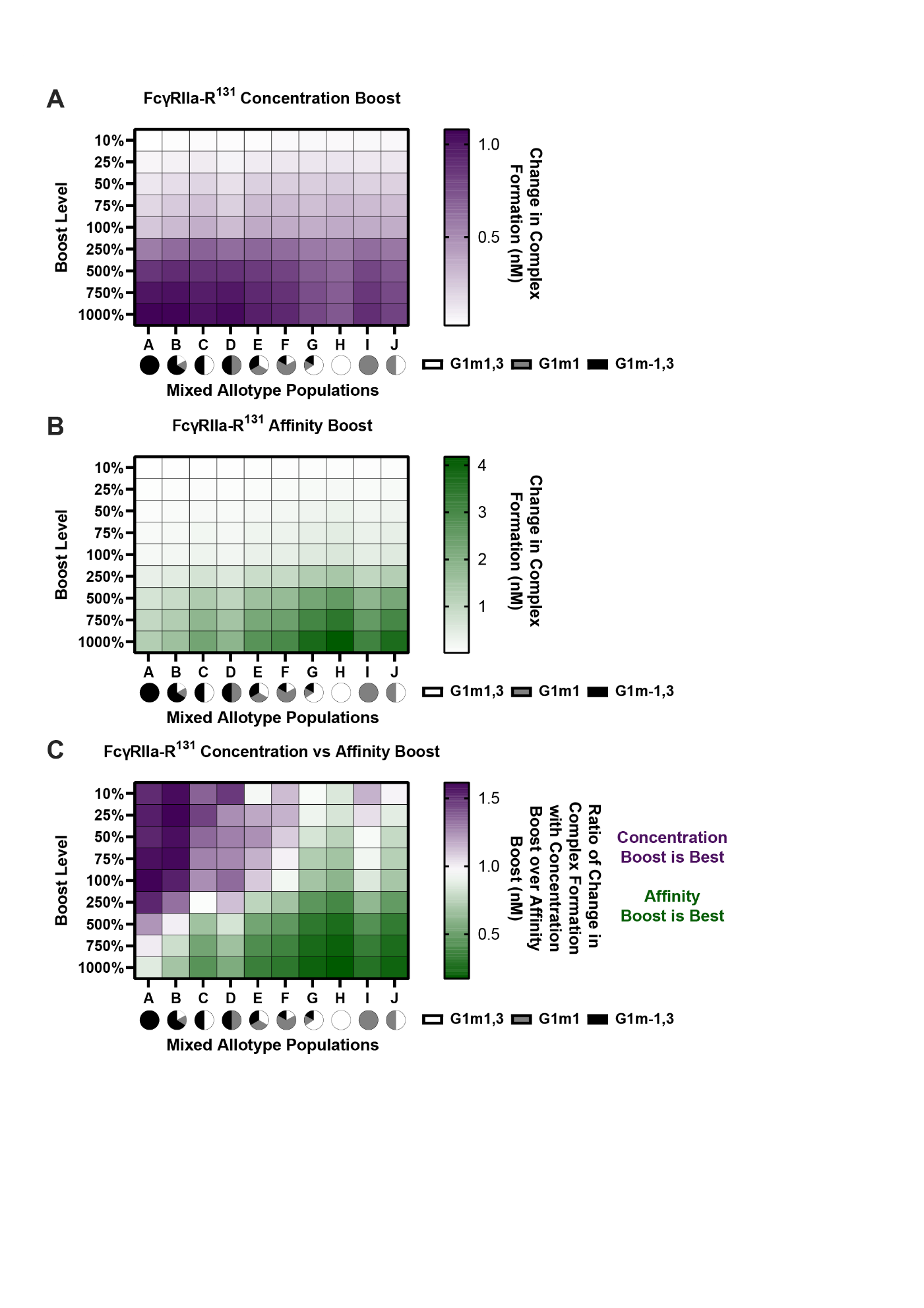
IgG1-FcγR, green).

**Supplementary Figure 4.** **In mixed allotype populations with FcRIIa-R^131^, the benefit of boosting IgG1 concentration vs. IgG1 affinity is dependent on the presence of the G1m-1,3 allotype.** (A) Boosting of initial IgG1 concentration in mixed allotype populations (G1m1,3, white; G1m1, gray; G1m-1,3 black) for FcγRIIa-R^131^. Color indicates predicted change in complex formation (B) Boosting of k_on_ IgG1- FcγR in mixed allotype populations (G1m1,3, white; G1m1, gray; G1m-1,3 black). Color indicates predicted change in complex formation (C) The ratio of median change in complex formation with a boost in IgG1 over median change in complex formation with a boost in k_on_ IgG1-FcγR at each boosting level. This ratio indicates which type of boost is predicted to be most effective for increasing complex formation (IgG1 concentration, purple; k_on_ IgG1-FcγR, green).
